# Discovery and small RNA profile of *Pecan mosaic-associated virus*, a novel potyvirus of pecan trees

**DOI:** 10.1038/srep26741

**Published:** 2016-05-26

**Authors:** Xiu Su, Shuai Fu, Yajuan Qian, Liqin Zhang, Yi Xu, Xueping Zhou

**Affiliations:** 1 State Key Laboratory of Rice Biology, Institute of Biotechnology, Zhejiang University, Hangzhou 310029, China; 2 The Nurturing Station for the State Key Laboratory of Subtropical Silviculture, Zhejiang Agriculture and Forestry University, Lin’an 311300, China; 3 State Key Laboratory for Biology of Plant Diseases and Insect Pests, Institute of Plant Protection, Chinese Academy of Agricultural Sciences, Beijing 100193, China

## Abstract

A novel potyvirus was discovered in pecan (*Carya illinoensis*) showing leaf mosaic symptom through the use of deep sequencing of small RNAs. The complete genome of this virus was determined to comprise of 9,310 nucleotides (nt), and shared 24.0% to 58.9% nucleotide similarities with that of other *Potyviridae* viruses. The genome was deduced to encode a single open reading frame (polyprotein) on the plus strand. Phylogenetic analysis based on the whole genome sequence and coat protein amino acid sequence showed that this virus is most closely related to *Lettuce mosaic virus*. Using electron microscopy, the typical *Potyvirus* filamentous particles were identified in infected pecan leaves with mosaic symptoms. Our results clearly show that this virus is a new member of the genus *Potyvirus* in the family *Potyviridae*. The virus is tentatively named Pecan mosaic-associated virus (PMaV). Additionally, profiling of the PMaV-derived small RNA (PMaV-sRNA) showed that the most abundant PMaV-sRNAs were 21-nt in length. There are several hotspots for small RNA production along the PMaV genome; two 21-nt PMaV-sRNAs starting at 811 nt and 610 nt of the minus-strand genome were highly repeated.

RNA silencing is an antiviral immunity and fundamental cellular mechanism that regulates gene expression through the production of small RNAs (sRNAs). Based on the finding that the viral small silencing RNAs (vsRNAs) can be overlapping in sequence and assembling into long contiguous fragments of the invading viral genome^1^, deep sequencing of small RNAs combined with bioinformatics analysis has been widely used for detection and identification of novel viruses, especially for viruses with low titer or without any pathogenic symptoms^2^,^3^,^4^,^5^.

*Potyviridae* is the largest and most economically important family of plant viruses, accounting for ~30% of the currently known plant viruses and causing significant losses in agricultural, pastoral, horticultural and ornamental crops^6^,^7^. *Potyvirus* is a genus in the family *Potyviridae*. Most potyviruses are transmitted by aphids in a non-persistent manner. The virions of potyviruses are non-enveloped with a flexuous and filamentous nucleocapsid of 680 ~ 900 nm long and 11 ~ 20 nm in diameter. The typical genomic RNA is a linear positive sense single-strand RNA ranging in size from 9,000 ~ 12,000 nucleotides. It contains a single long ORF, which encodes a large polyprotein that is cleaved into 11 mature proteins including P3N-PIPO (P3 N-terminus-pretty interesting potyvirus ORF)^8^, which is produced by ribosomal frameshift during translation or transcriptional slippage during RNA synthesis in the P3 cistron^9^. And recently, a novel translational frameshift gene product named P1N-PISPO (pretty interesting sweetpotato potyvirus ORF) was discovered in sweet potato feathery mottle virus (SPFMV) infected plants^10^, and functions as an RNA silencing suppressor^11^,^12^.

Pecan (*Carya illinoensis*) is native to central and southern North America. It is an excellent multipurpose tree, providing a source of nutritious edible nuts, furniture-grade wood, and aesthetic value for the landscape. Pecan nuts are ranked highest in antioxidant capacitance and are enriched with many nutrients, minerals, and vitamins that are essential for optimum health. Pecans have been extensively cultivated in many countries for their commercial value. In China, pecan is increasingly being grown in many regions, including Hebei, Henan, Jiangsu, Fujian and Zhejiang provinces. Diseases affecting pecans are mainly caused by bacteria, fungi, nematodes, and physiological problems. No viruses have been reported to occur in pecans until now. During our survey of plant viruses of woody plants in southeast China, we found several pecan trees which showed typical symptoms of plant virus infection, with systemic mosaic on leaves. Further experiments were performed and finally a novel potyvirus was identified from these pecan trees. The virus is tentatively named Pecan mosaic-associated viru*s* (PMaV). Furthermore, small RNA profiles of this virus suggest that the RNA silencing-mediated antiviral immunity mechanism is also applicable to pecan. To our knowledge, this the first viral disease found in pecan.

## Results

### Symptom of PMaV infected pecan trees

**Figure 1:**
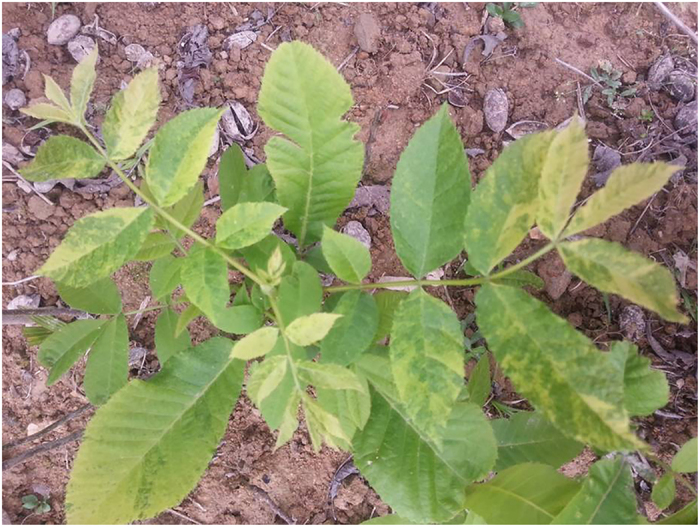
The mosaic symptom of pecan leaves.

Mosaic symptom was observed on the leaves of pecan trees during a field survey in pecan orchards. These plants show an overall lighter color along with mosaic patterns on some leaves, especially on the younger leave (Fig. 1). Then, the leaves with symptom were used for RNA isolation.

### Deep sequencing and small RNA characterization

A total of 17,832,459 raw reads was obtained, which contained 17,297,837 clean reads with length between 18 and 30 nt after filtering. All these small RNAs were assembled into contigs using Velvet software with k-mer value of 17^13^. After searching against the GenBank database, we found 35 contigs which shared some similarities with the genome sequences of other potyviruses, such as turnip mosaic virus (TuMV), lettuce mosaic virus (LMV), SPFMV and plum pox virus (PPV), suggesting that one or more potyviruses were present in our sample.

### Complete genome characterization

**Figure 2:**
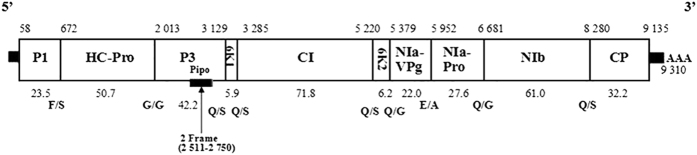
Genome organization of PMaV. Dash areas represent 5′ UTR and 3′ UTR; the numbers above the figure represent the number of nucleotide of each proteins; the numbers below the figure represent the molecular mass of each proteins; the arrow represents protein PIPO in P3 protein zone, which is located at 2,511–2,750 nt of the PMaV genome; the 9 cleavage sites on this polypeptide also below the figure.

Based on the contig sequences, the viral genome was obtained through several RT-PCR amplifications covering all gaps. After sequencing, we ensured that only one virus existed in our sample. RACE-PCR was performed to obtain the 5′- and 3′-termini sequences. As shown in Fig. 2, this virus was determined to comprise 9,310 nucleotides (nt) with a GC content of 39.15%. BLASTN search results showed that the virus was most similar to viruses of the genus *Potyvirus* (family *Potyviridae*). All thirty-six available complete genome sequences of *Potyviridae* viruses from GenBank were retrieved, and pairwised genome similarity comparison was performed. As a result (Table 1), the full-length nucleotide sequence similarities ranged from 24.0% (wheat yellow mosaic virus, WYMV) to 58.9% (LMV), and full-length amino acid (aa) sequence similarities from 19.7% (WYMV) to 53.6% (LMV). PMaV CP had the highest sequence identity with LMV on aa level (69.5%), followed by TuMV with 69% aa similarity. On CP nt level, PMaV CP had the highest sequence identity with TuMV (61.2%). All similarities are below the established threshold used to discriminate species of the genus *Potyvirus*^14^. Our results clearly demonstrated that this virus is a new species in the genus *Potyvirus*.

### Genome structure analysis

The whole genomic sequence of this virus is 9,310 nt long, excluding the 3′ poly (A) tail, and has been submitted to the GenBank database under the accession number KT633868. The genomic organization of this virus is very similar with other potyviruses, consisting of a single open reading frame (ORF) (Fig. 2). The ORF starts at the first in-frame AUG (58–60 nt) and ends with a UAG (9136–9138 nt) termination codon; a polyprotein of 3,026 amino acids with a calculated molecular mass of 342.8 kDa was deduced to be translated. The nine putative protease cleavage sites were identified in the polyprotein (Fig. 2), that were predicted to generate eleven putative mature proteins, including a small P3N-PIPO protein was predicted to be expressed by polymerase slippage mechanism from the P3 ORF.

Proteinase P1 comprises 205 amino acids (23.5 kDa) encoded by nucleotides located at position 58–672 nt. The mature protein is predicted to be cleaved at the sequence WRGF/S, which is cut by P1 serine proteinase. P1 of this virus has a motif ^143^GWSG^146^ which is typical of potyviruses. The helper component proteinase (HC-Pro) comprises 447 residues (50.7 kDa) encoded by nucleotides located at position 673–2,013 nt, and is cleaved at the sequence YKVG/G, in agreement with the classical motif YXVG/G. The ^375^FRNK^378^ box, which is involved in RNA silencing suppression and associated with symptom development, is present^15^. Motifs of ^246^RITC^249^ and ^504^PTK^506^, which are associated with aphid transmission^16^ are also included. The P3 protein (2,014–3,129 nt) is composed of 372 residues (42.2 kDa), and is cleaved at the site ITLQ/S. An ORF for putative P3N-PIPO protein was identified that include the frame shift sequence ^2511^GA-AAAAA^2750^ and with a UAG stop codon at the position 2,751 nt. The cylindrical inclusion (CI) protein comprises 645 residues (71.8 kDa) encoded by nucleotides located at position 3,286–5,220 nt, and is cleaved at the site MTFQ/S. CI contains the ^1161^GAVGSGKST^1169^ and ^1250^DECH^1253^ motifs typical of the helicases of the superfamily 2^17^, and three other conserved motifs (^1423^GERIQRLGRVGR^1434^, ^1380^ATNIIENGVTL^1390^ and ^1277^KVSATPP^1283^) whose functions are not clear. Nuclear inclusion body ‘a’ protein (NIa) includes the VPg (NIa-VPg) and NIa protein (NIa-Pro). Virus genome-linked protein (VPg) is a basic protein with 191 amino acids (22.2 kDa) encoded by nucleotides located at position 5,380–5,952 nt, and is cleaved at the sequence IQFE/A. Nuclear inclusion protein (NIa-Pro, 5,953–6,681 nt) comprises 243 residues (27.6 kDa), and is cleaved at the sequence MKFQ/G. NIa-Pro has cysteine protease active site which is responsible for cleavage at the P3/6K1, 6K1/CI, CI/6K2, 6K2/VPg, VPg/NIa-Pro, NIa-Pro/NIb, NIb/CP catalytic site with the consensus ^2011^H-34X-D-67X-G-X-C-G-14X-H^2132^. Nuclear inclusion body ‘b’ protein (NIb) comprises 533 residues (61.0 kDa) encoded by nucleotides located at position 6,682–8,280, and was cleaved at the site ASAQ/S. It also contains the typical RdRp motif GDD (^2515^GNNSGQPSTVVDNTLMVILAIVYSLIKLGHPYRTHKDIIKYFVNGDD^2561^). Finally, the CP (8,281–9,129 nt) comprises 285 residues (32.2 kDa), and also contains the highly conserved ^2735^DAG^2737^ motif involved in aphid transmission^18^.

### Phylogenetic and recombination analysis

**Figure 3:**
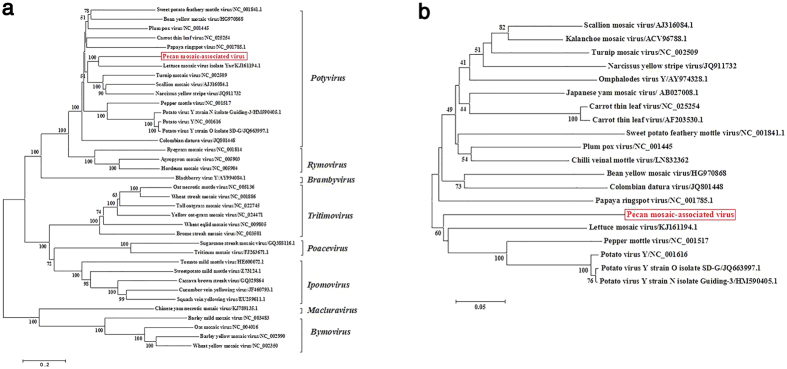
Neighbor-joining phylogenetic tree of *Potyviridae* viruses based on full genomic sequences (**a**) and CP amino acid sequences (**b**). Branch numbers represent the percent similarity in the bootstrap values out of 1000 replicates.

For phylogenetic analysis, multiple-alignments were first performed using the Muscle program^19^. Phylogenetic relationships between PMaV and other potyviruses were estimated based on their full genome sequences (Fig. 3a) and the amino-acid sequences of their coat proteins (Fig. 3b). The two phylogenetic trees had similar topologies, and both phylogenetic trees showed that this virus is most closely related to LMV. Based on the full genome sequence, this virus clusters with LMV, while on the amino acid sequence of CP, it forms a distinct branch adjacent to LMV.

Recombination is a dominant feature of potyvirus evolution. To see whether or not the novel PMaV was generated by recombination, recombination analysis was performed using two different programs (TOPALi and RDP). No recombination events were detected, independently of the data set used for the analysis (data not shown).

### Detection of PMaV particle

**Figure 4:**
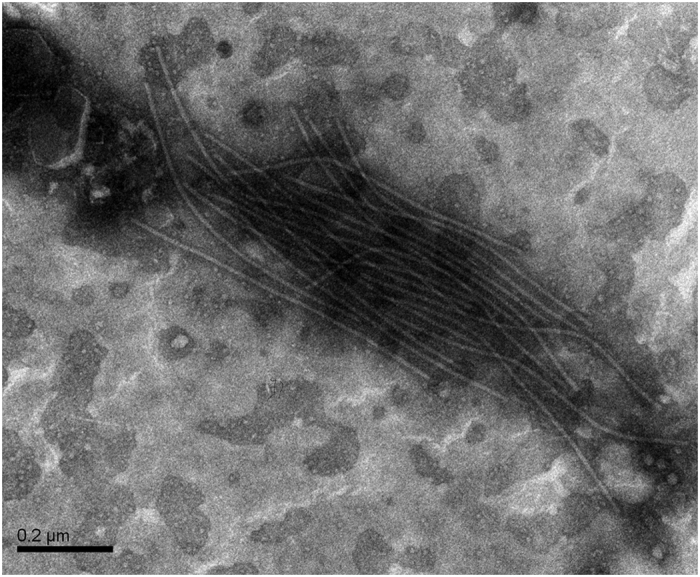
Filamentous virion in a leaf dip from infected pecan leaves with mosaic symptom. The bar represents 200 nm.

Using electron microscopy, the typical filamentous *Potyvirus* particles were found in infected pecan leaves with mosaic symptoms (Fig. 4), but were absent in healthy, control pecan leaves. The filamentous particle appeared to be non-enveloped and flexuous, measuring 680 ~ 800 nm long and 12 ~ 15 nm in diameter (Fig. 4). This result, together with sequence and structure composition, indicates that the virus identified in this study is a new member of the genus *Potyvirus* within the family *Potyviridae*.

### Host range determination

An experimental host range for PMaV was determined by mechanical inoculation of two plant species in *Solanaceae* family that are considered as common virus indicator hosts. No evidence of infection was detected in any of the tested *Nicotiana tabacum*, or *N. benthamiana* (data not shown). This result is in line with the fact that most potyviruses have narrow host ranges.

### Profiling of PMaV-derived small RNAs

**Figure 5:**
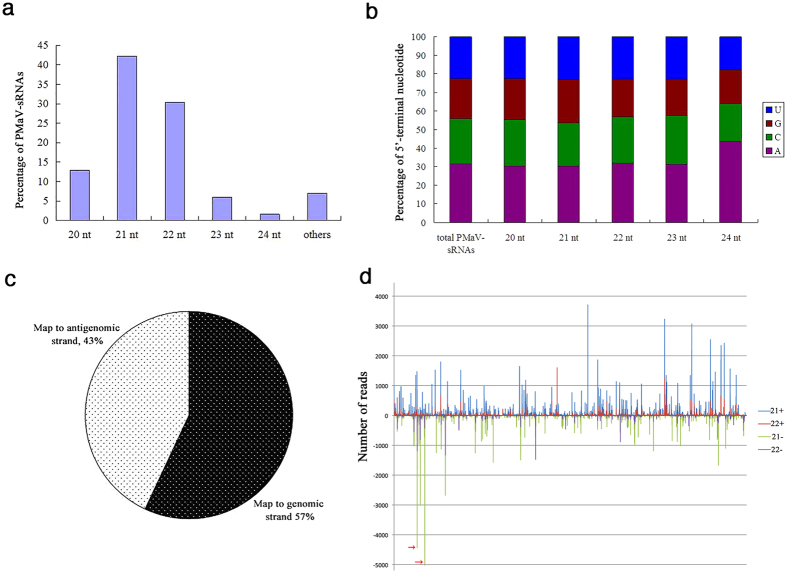
Small RNA profiling of PMaV from infected pecan leaves. (**a**) Bar graph showing the mapped PMaV-sRNA size distributions; (**b**) Relative frequency of 5′-terminal nucleotide for 20 to 24-nt PMaV-sRNAs; (**c**) Statistical analysis of PMaV-sRNAs mapped to the genomic or antigenomic sequences; (**d**) Genome-wide mapping of PMaV-sRNAs. The horizontal axis represents the relative position along the PMaV genome. The vertical axis represents the number of PMaV-sRNAs reads mapped to the PMaV genomic (+) or antigenomic (−) sequences. Arrows represent 811 nt and 610 nt of the minus-genomic RNA sequence.

A total of 628, 030 sRNAs can be mapped to the PMaV genomic sequence, which account for 3.77% of the total sRNAs. The size classes of PMaV-derived small RNAs (PMaV-sRNAs) were mostly 21 to 24-nt in length; among them the 21-nt class is most prominent, accounting for 42.22% of the total PMaV-sRNAs, followed by the 22-nt sRNAs (Fig. 5a). In *Arabidopsis*, the 5′-terminal nucleotide partially determines the preference of sRNAs for AGO proteins. Therefore, the distribution of 5′-terminal nucleotides was determined for the sequenced PMaV-sRNAs (Fig. 5b). For all four types of PMaV-sRNAs characterized (i.e., 20-, 21-, 22- and 23-nt), there is no significant difference for 5′-terminal nucleotide preference. In contrast, the 24-nt PMaV-sRNAs primarily contained adenine at the 5′-terminus (44%), indicating that they might be loaded into pecan AGO2 and AGO4 homologues, and may participate in transcriptional silencing for host transposons and repeated sequences.

Polarity analysis of redundant PMaV-sRNAs showed that they were derived slightly more often from the positive-sense genomic sequence (57%) than the antisense genomic sequence (43%) (Fig. 5c). Further analysis of those 21 and 22-nt PMaV-sRNAs showed that, within the PMaV genome, the distribution peaks representing the 21-nt PMaV-sRNAs almost overlapped perfectly with the peaks representing the 22-nt PMaV-sRNAs (Fig. 5d). The PMaV-sRNAs from both strands were discontinuous and unevenly distributed along the viral genome (Fig. 5d). However, three significant hotspots of small RNAs were identified along the PMaV genome. Among them, two are from the positive-sense genome, and are located in the CI-encoding region and CP-encoding region, respectively. The other hotspot is derived from the minus-strand genome, located in the regions encoding the P1 and HC-Pro proteins (bases from 590–811). In the region, two 21-nt PMaV-sRNAs, beginning at 811 nt and 610 nt, were highly repeated (Fig. 5d).

## Discussion

Pecan tree are increasingly being grown commercially all over the world because pecan nuts supply many valuable nutrients for a healthy diet. At present, there are no reported viral diseases found in pecans. In our research, we isolated and identified a new virus, tentatively named PMaV, from pecan in Lin’an, Zhejiang province, China using small RNA deep sequencing technology. The complete genomic sequence of PMaV was cloned and sequenced. The full-length nucleotide sequence only shared 58.9% similarity with its closest relative, LMV. Furthermore, using electron microscopy, we identified the typical *Potyvirus* filamentous particles in infected pecan leaves presenting mosaic symptoms. As a whole, these results support our finding that PMaV is a new species in the genus *Potyvirus*. During our investigation, we observed very severe mosaic symptoms in PMaV infected leaves; however, its pathogenicity in pecan is still being assessed. Also, this virus cannot be mechanically inoculated onto the two common hosts reported for other members of the family *Potyviridae*, indicating that PMaV may also have narrow host ranges. Other plants nearby the pecan trees are still under testing.

To date, several potyviruses have been reported as targets of the RNA silencing machinery of various herbaceous host plants. However, there is little information on this mechanism in woody plants. Here, profiling of small RNAs from PMaV-infected pecan leaves was performed. The presence of large quantities of PMaV-sRNAs (3.77% of total sRNAs) indicates that RNA silencing-mediated antiviral immunity is also utilized by pecan. In contrast to other potyvirus-derived small RNAs in herbaceous plants, which display a clear preference for adenine and uracil bases at the 5′-end^20^,^21^, there is no significant difference in the 5′-terminal nucleotide preference in PMaV-sRNAs from pecan (Fig. 5b). Although polarity analysis of redundant PMaV-sRNAs showed that they were derived slightly more often from the positive-sense genomic sequence (57%) than the antisense genomic sequence (43%) (Fig. 5c), we still found that two 21 nt sRNAs, starting at 811 nt and 610 nt of the minus-genomic sequence, were highly repeated (Fig. 5d). This is unexpected, because for positive-strand RNA viruses, the genomic-strand viral RNAs (plus-strand) are typically more abundant than the complementary-strand RNAs (minus-strand genome). The secondary structure of RNA should not be responsible for this difference, because there is no difference in the secondary structure between the two strands of RNA. To better understand this phenomenon, polarity analysis of non-redundant PMaV-sRNAs was performed; we found an almost perfect match of hotspot distribution between the plus- and minus-genome (Supplementary Fig. S1). Therefore, our results give some clue as to the PMaV-sRNAs’ biogenesis: PMaV-derived nonredundant sRNAs originate from viral dsRNAs and subsequent biogenesis of redundant vsRNAs might be a combination of primary vsRNAs from dsRNAs and secondary vsRNAs from host RDRs. RDRs responsible for generating secondary vsRNAs has been shown to preferentially amplify vsRNAs from distinct hot spots within the viral genome in plants^22^. Further research is needed to unveil the function of this kind of minus-genome derived PMaV-sRNA.

There are still many questions regarding PMaV epidemiology that need to be answered. First, pecan trees are native to central and southern North America, but we still do not know the geographic origin of the virus. Second, although most potyviruses in nature are transmitted by aphids, we do not yet know the mode of PMaV transmission. There are several species of aphids that inhabit pecan trees, such as the black margined aphid (*Monellia caryella*) and the yellow pecan aphid (*Monelliopsis pecanis*), but we do not know whether these aphids can transmit PMaV. In contrast to herbaceous plants, woody plants are usually bred by grafting; whether or not this virus can be transmitted to pecan tree through grafting is currently under investigation. Our study, identifying a novel potyvirus that infects pecan trees, will assist in the development of molecular diagnosis of this virus in pecan trees all over the world.

## Methods

### RNA isolation and deep sequencing

Total RNA was extracted using Trizol reagent (Invitrogen, Carlsbad, USA) following the manufacturer’s instructions. The small RNA library was constructed as described^23^. Briefly, low molecular mass (LMW) RNA in the total RNA samples was enriched using PEG (molecular mass 8000) and NaCl (5M). LMW RNAs (sRNAs) were sequentially ligated to a 3′ and a 5′ adapter. After ligation, sRNAs were purified by electrophoresis using a 15% denaturing PAGE. The final purified ligation products were reverse transcribed into cDNAs using the Superscript III reverse transcriptase (Invitrogen, Carlsbad, CA, USA). The first strand cDNAs were PCR amplified using the Taq polymerase (Roche Applied Science, Basel, Switzerland) and DNA fragments were purified for high-throughput sequencing using the Solexa platform (BGI, Shenzhen, China).

### Genome assembly and sequence analysis

Assembled contigs were scanned against the GenBank database using BLASTN (http://www.ncbi.nlm.nih.gov/). Then the whole viral genome was obtained through several RT-PCR amplifications covering all the gaps, using a battery of sense and antisense-specific primers designed based on the contig sequences. The 5′- and 3′-terminal sequences were determined using 5′/3′ RACE PCR (Clontech, Palo Alto, CA, USA). The primer sequences were shown in Supplementary Table S1. PCR was performed in a total volume of 50 μL with Bio-rad thermal cycler S1000^TM^ (Life Science, Foster City, CA, USA) with Phusion^®^ high-fidelity DNA polymerase (New England BioLabs, Ipswich, MA, USA). The amplified fragment was recovered with the AxyPrep^TM^ DNA Gel Extraction Kit (Axygen Biosciences, Union City, CA, USA) and cloned using the ZeroBack Fast Ligation Kit (Tiangen, Beijing, China). The positive colonies were further confirmed by DNA sequencing.

The obtained sequences were assembled into a contiguous sequence at a standard of ≥99.9% similarity at each overlapping region using DNAStar 7.01 package (DNASTAR Inc., Madison, USA). The putative cleavage sites and conserved domains of the putative functional proteins in the PMaV polyprotein were determined by comparison of its own sequence with other potyviruses^14^.

### Phylogenetic and recombination analysis

Thirty-six whole genome sequences and twenty CP amino-acid sequences of representative recognized members of each of the eight genera comprising the family *Potyviridae* were retrieved from NCBI (http://www.ncbi.nlm.nih.gov/). Phylogenetic trees were constructed by the Neighbor-joining method using MEGA 6.06 software^24^. Recombination events were searched within the PMaV genome or in the CP genes, using two distinct data sets: (i) complete genome sequences representing the major lineages of potyviruses; (ii) all CP sequences deposited in GenBank corresponding to potyviruses; the analysis was performed with TOPALi v2.5 and RDP v4.43. Recombination analyses were performed using default settings for each detection program and a Bonferroni corrected *P*-value cut-off of 0.05.

### Electron microscopy

Samples from pecan leaves with mosaic symptoms and non-mosaic leaves were prepared for transmission electron microscopy (TEM). The samples were grinded with 0.5 ml ddH_2_O in a mortar, dipped the grid in the serosity for about 10 seconds, then dry with filter paper. The grid was covered with a drop of 2% tungsten phosphate staining solution (pH 7.0) for about 20 seconds, and the copper grid was dried before EM observation.

### Bioinformatic analyses of sRNA sequences

After removal of the adapter sequences and low quality reads by in-house Perl script, the resulting clean reads were then assembled into larger contigs using Velvet 0.7.31 with the k-mer value of 17^13^. For hotspot analysis, PMaV-derived small RNAs (PMaV-sRNAs) were extracted by alignment with the PMaV genome via bowtie1 allowing zero mismatch (version 0.12.8, Langmead, 2009), and then PMaV-sRNA hotspots along the PMaV genome were generated by executing in-house Perl scripts.

## Additional Information

**Accession codes:** Sequencing data were deposited in NCBI GenBank database with the accession number KT633868.

**How to cite this article**: Su, X. *et al.* Discovery and small RNA profile of *Pecan mosaic-associated virus*, a novel potyvirus of pecan trees. *Sci. Rep.* **6**, 26741; doi: 10.1038/srep26741 (2016).

## Supplementary Material

Supplementary Information

## Figures and Tables

**Table 1:** Percent nucleotide (nt) and amino acid (aa) sequence identities of pecan mosaic-associated virus to ten other potyviruses.

Virus^a^	ScMV	LMV	PRV	PPV	PMV	TuMV	PVY-O	PVY-N	SPFMV	WYMV
Genomic sequence (nt)	55.8	58.9	52.6	54.6	53.0	55.4	25.1	51.8	54.0	24.0
Whole polyprotein (aa)	50.4	53.6	46.2	52.0	47.0	50.7	47.5	47.3	49.9	19.7
CP (nt)	59.0	57.4	59.0	56.0	59.4	61.2	27.1	60.7	55.6	37.8
CP (aa)	66.2	69.5	63.8	63.4	66.7	69.0	65.7	66.2	60.1	9.9

^a^Sequences obtained from GenBank: scallion mosaic virus (ScMV, AJ316084.1), lettuce mosaic virus (LMV, KJ161194.1), papaya ringspot virus (PRV, NC_001785.1), plum pox virus (PPV, NC_001445), pepper mottle virus (PMV, NC_001517), turnip mosaic virus (TuMV, NC_002509), potato virus Y strain O isolate (PVY-O, JQ663997.1), potato virus Y strain N isolate (PVY-N, HM590405.1), sweet potato feathery mottle virus (SPFMV, NC_001841.1) and wheat yellow mosaic virus (WYMV, NC_002350).
