# Supplementary material for: Discovery and small RNA profile of Pecan mosaic-associated virus, a novel potyvirus of pecan trees
Source: Sci Rep. 2016 May 26;6:26741. doi: 10.1038/srep26741 (PMC4880897; doi:10.1038/srep26741)

Discovery and small RNA profile of *Pecan mosaic-associated virus*, a novel potyvirus of pecan trees

Xiu Su[1,2†], Shuai Fu[1†], Yajuan Qian[1], Liqin Zhang[2], Yi Xu[1*] and Xueping Zhou[1,3*]

[1] State Key Laboratory of Rice Biology, Institute of Biotechnology, Zhejiang University, Hangzhou 310029, China;

[2] The Nurturing Station for the State Key Laboratory of Subtropical Silviculture, Zhejiang Agriculture and Forestry University, Lin'an 311300, China;

[3] State Key Laboratory for Biology of Plant Diseases and Insect Pests, Institute of Plant Protection, Chinese Academy of Agricultural Sciences, Beijing 100193, China

*Author to whom correspondence should be addressed. Email: xuyiqdpd@zju.edu.cn or; zzhou@zju.edu.cn;

† These authors contributed equally to this paper.

Supplementary Table S1: Oligonucleotide primers used in this study

Primer name	Position[a]	Nucleotide sequence (5'-3')
5' UPM-Long[b]		CTAATACGACTCACTATAGGGCAAGCAGTGGTAT-CAACGCAGAGT
5' UPM-Short[b]		CTAATACGACTCACTATAGGGC
5'-R1	1068-1095	GAGTTCACTCGACGATAACTCGCCACCC
5'-R2	862-889	CGCTCATGACGTGTGAGCTTTAGATCG
ND104-F	820-843	ATTGGAGCACAGAGAACCAAAAGC
ND240-R	1848-1873	GGTGATCAACTAAAATCCTTGGTAGC
ND240-F	1444-1469	GAACCTGTCGAAGAGCAAAAACTAAC
ND649-R	3919-3944	TCCACTGGATATTGAGTCTGGAATTC
ND649-F	3800-3825	TCTTTGATGAGTGCCATGTCATAGAC
ND159-R	4573-4595	ATCGAACCATCATAGCGAACCAG
ND159-F	4123-4145	GGTCGAACTATGAAGAGTGGTAG
ND228-R	6532-6554	CTCTGCCAGTTTAGAGATTCAGG
ND228-F	6019-6041	AACGAGACCGATGGACAATTCAG
ND78-R	7273-7296	ATCCACACATACCTTTCCTCCCAG
ND78-F	6580-6605	ATTGTTTGGGGAAGTCTAGAGTTGAG
ND85-R	8222-8246	GTCTCATTAGCTTGGAAGGTGATAC
ND85-F	7734-7757	GGATGATCTCTTAATCGCAGTCAG
ND188-F	8969-8989	ACAGAGCAAGAGAAGCCCACC
ND188-R	9287-9310	TCTCTTAGCTCCTGGCAATCACAG
3'-F1	8969-8989	ACAGAGCAAGAGAAGCCCACC
3'-F2	9287-9310	CTGTGATTGCCAGGAGCTAAGAGA
3'RACE-R[c]		GCGAGCACAGAATTAATACGAC

[a] Primer positions on the complete sequence of PaMV.

[b] Anchored primers, used for 5'-RACE.

[c] Degenerate primers, designed from the conserved sequences of PVY isolates.

Supplementary Figure 1 Genome-wide mapping of PMaV-sRNAs along PMaV genome using non-redundant PMaV-sRNAs. The horizontal axis represents the relative position along the PMaV genome. The vertical axis represents the number of PMaV-sRNAs reads mapped to the PMaV genomic (+) or antigenomic (-) sequences.

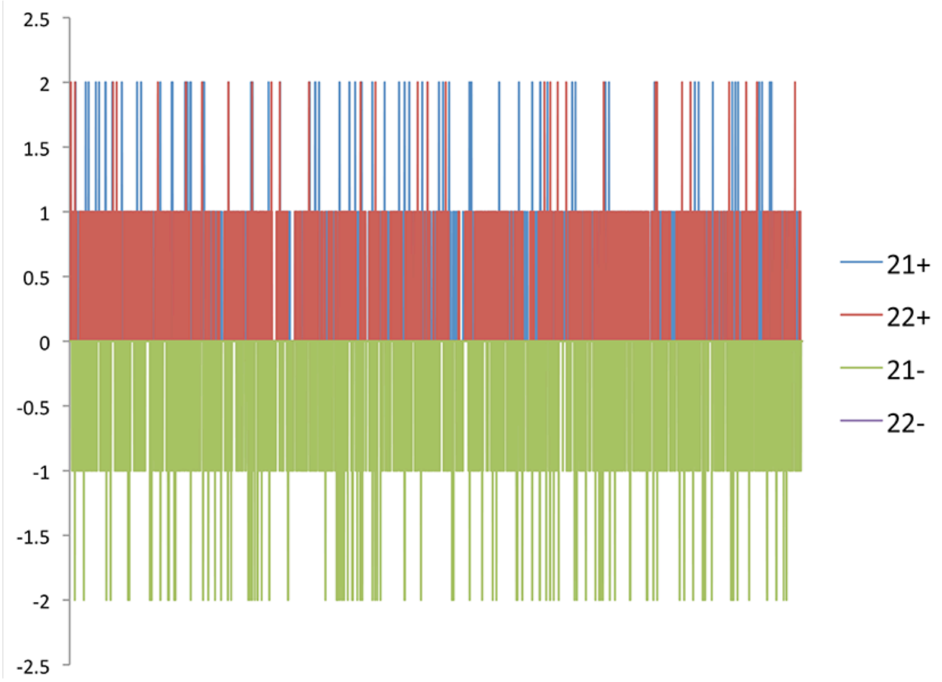

Supplement: Supplementary Information [file srep26741-s1.pdf]
